# Exploring the status of and demand for palliative day-care clinics and day hospices in Germany: a protocol for a mixed-methods study

**DOI:** 10.1186/s12904-021-00792-5

**Published:** 2021-06-24

**Authors:** Franziska A. Herbst, Stephanie Stiel, Kathrin Damm, Lea de Jong, Jona T. Stahmeyer, Nils Schneider

**Affiliations:** 1 Institute for General Practice, Hannover Medical School, Carl-Neuberg-Strasse 1, 30625 Hannover, Germany; 2 Center for Health Economics Research Hannover (CHERH), Leibniz University Hannover, Otto-Brenner-Straße 7, 30159 Hannover, Germany; 3 Health Services Research Unit, AOK Niedersachsen, Hildesheimer Straße 273, 30519 Hannover, Germany

**Keywords:** palliative care, hospice care, day-care, medical, adult day-care centers, patient preference, end of life care, palliative day-care clinic, day hospice, health care planning

## Abstract

**Background:**

To date, the establishment and development of palliative day-care clinics and day hospices in Germany have been completely unsystematic. Research is needed to gain insight into these services and to ensure their accessibility and quality. Accordingly, the ABPATITE research project aims at: (1) identifying the characteristics of palliative day-care clinics and day hospices in Germany, (2) determining demand and preferences for these services, and (3) proposing recommendations (with expert agreement) for the needs-based establishment and development of these services.

**Methods:**

The research is a multi-perspective, prospective, observational study following a mixed-methods approach across three study phases. In phase 1a, qualitative expert interviews will be conducted to capture the facility-related characteristics of palliative day-care clinics and day hospices in Germany; the results will feed into a questionnaire sent to all such institutions identified nationwide. In phase 1b, a questionnaire will be sent to local statutory health insurance providers, to gain insight into their contracts and accounting and remuneration models. In phase 2a, a service preference survey will be conducted with patients and family caregivers. In phase 2b, semi-structured interviews with management staff will explore the factors that promote and hinder the provision of service. In phase 2c, the external perspective will be surveyed via focus groups with local actors involved in hospice and palliative care. In phase 3a, focus groups with representatives from relevant areas will be conducted to develop recommendations. Finally, in phase 3b, recommendations will be agreed upon through a Delphi survey.

**Discussion:**

The empirically developed recommendations should enable the establishment and development of day hospices and palliative day-care clinics in Germany to be better managed, more oriented to actual demand, and more effectively integrated into wider health care services. Importantly, the findings are expected to optimize the overall development of hospice and palliative care services.

**Trial registration:**

The study was prospectively registered in the German Clinical Trials Register (Deutsches Register Klinischer Studien) (Registration N° DRKS00021446; date of registration: April 20, 2020). The study is searchable under the International Clinical Trials Registry Platform Search Portal of the World Health Organization, under the German Clinical Trials Register number.

## Background

In Germany, trans-sectoral inpatient and ambulatory hospice and palliative care services for people with incurable, progressive illnesses and limited lifespans have significantly advanced in recent years, in both specialized and general sectors. More than likely, this is due to the socio-political relevance of and support for end of life care in Germany, where inpatient hospices, palliative care units, ambulatory hospice services, and specialized palliative home care services are widely established.

The Hospice and Palliative Care Act 2015 has placed a greater focus on strengthening and expanding general palliative care in Germany [1]. Specifically, this dynamic law aims at establishing needs-oriented, comprehensive care services in both urban centers and economically weak and rural regions, as well as improving access to all hospice and palliative care services, particularly for immigrants and members of socially disadvantaged groups (e.g. prisoners). This promotion of a needs-based, patient-oriented approach to hospice and palliative care also applies to palliative day-care clinics and day hospices [2]. While these services are not necessarily defined by social law in Germany, they must still adhere to regulations in order to successfully claim costs back from statutory health insurance providers. Day-care services are often linked to institutions such as inpatient hospices and palliative care units or hospitals; however, they may also operate independently. The primary aims of these day-care institutions, in which patients spend up to five days per week, are maintaining patients’ autonomy, improving patients’ quality of life, and supporting family caregivers by granting them “respite care” [3].

To date, the establishment and development of palliative day-care clinics and day hospices in Germany have been completely unsystematic. Thus, existing institutions may have implemented—and may continue to implement—insular solutions that are not coordinated with other institutions or with regional health care services, or indeed with the general health care services covered by statutory health insurance providers. For this reason, patients at their end of life may find that these institutions are not sufficiently integrated into the German health care system. In Germany, structural standards and quality indices for palliative day-care do not exist, and the label of “day hospice” is applied to practices that are very heterogeneous, in terms of services offered. This can generate confusion among patients and family caregivers, making it difficult for them to choose an adequate and needs-oriented form of care. However, standards do exist for services financed by statutory health insurance providers, and these standards ensure a high quality of care [4].

### Investigating the status of and demand for palliative day-care clinics and day hospices

In 2010, the European Association for Palliative Care estimated that one palliative day-care clinic or day hospice was needed for a catchment area of 150,000 residents [5]. More recent surveys of demand in Germany have investigated palliative day-care clinics and day hospices only marginally, or not at all [6–8]. Internationally, mostly qualitative interview studies and quantitative surveys and analyses of patient records show a high level of patient satisfaction with the quality of care received at palliative day-care clinics and day hospices, relating to the opportunities for social participation offered by these institutions and their maintenance of quality of life [9, 10]. However, systematic findings from controlled studies of high methodological quality are lacking, particularly in relation to the effectiveness of these end of life institutions in: improving symptoms, providing inter-sectoral continuity of care, preventing inpatient hospital admissions and nursing home or hospice admissions, and delivering cost savings across the course of a disease [11, 12]. Notwithstanding the scientific evidence for the need and effectiveness of day-care services in Germany, initiatives to establish palliative day-care clinics and day hospices are evolving [13–22].

### Study aims

Scientific research is needed to provide an overview of current hospice and palliative day-care services in Germany; to increase the transparency of these services over the long term; and to ensure the accessibility and quality of these services. In response to this need, the ABPATITE consortium study aims at answering the following questions, across three phases:

Phase 1: What palliative day-care clinics and day hospices are operating or in development in Germany, and what are the characteristics of these facilities?

Phase 2: What is the demand for palliative day-care clinics and day hospices in Germany, as assessed by health care professionals, stakeholders, and representatives, and what are the service preferences of patients and family caregivers?

Phase 3: What recommendations can be determined and agreed upon for the needs-based establishment and development of day hospices and palliative day-care clinics in Germany?

### Aims of the study protocol

The present research is a mixed-methods study involving qualitative and quantitative methods and participatory aspects. The project aims at making an original contribution to the palliative care literature by increasing the transparency of palliative day-care clinics and day hospices in Germany. Publication of the study protocol represents the first step in this process. In their previous Dy@EoL project [23], the authors experienced that recruitment partners and scientists studying similar topics were interested in consulting the study protocol to obtain an overview of the project. Furthermore, the processes of establishing and developing institutions and measuring status and demand in palliative day-care clinics and day hospices are not necessarily straightforward. The present study protocol is designed to shed light on these processes. In addition, the study protocol addresses ethical considerations, data security, and the dissemination and implementation of the study results. Finally, the study design for systematic research on palliative day-care clinics and day hospices is of relevance to the scientific community.

## Methods and design

The present multi-perspective, prospective, observational study follows a mixed-methods approach, spread across three research phases. The study protocol adheres to STROBE guidelines [24].

In phase 1a, palliative day-care clinics and day hospices in Germany are being identified through national online databases of hospices and palliative care providers [25, 26] and general Internet searches. Exploratory, qualitative expert interviews are being conducted (currently underway) with facility managers to capture relevant facility-related characteristics. These results will be used to develop a standardized quantitative questionnaire that will be sent to all palliative day-care clinics and day-hospices identified nationwide.

In phase 1b, a quantitative, standardized instrument will be developed in cooperation with a local statutory health insurance provider (AOK Lower Saxony) to identify and record all day hospices and palliative day-care clinics that are contracts with the statutory health insurance funds. The instrument will not only record the contractual partners, but it will also capture contractual terms relating to remuneration and services provided, as well as the number of patients treated in these facilities. Further, the number of claims and approvals filed by the day hospices and palliative day-care clinics will be collected.

In phase 2a, a preference survey about hospice and palliative care will be administered to patients with a potentially palliative course of illness and their family caregivers. The aim of this quantitative step will be to determine the preferences of patients and their family caregivers regarding palliative care services they may draw on in the future. The questionnaire will contain a case description of a patient in palliative care, as well as a discrete choice experiment (DCE) [27, 28]. In the DCE, participants will be asked to choose between two alternative palliative care situations. From the DCE data, conclusions will be drawn about the perceived importance of individual care characteristics. The questionnaire will also capture patients’ socio-demographic and medical data (related specifically to their disease), as well as data on family caregivers’ willingness or capacity to provide care. A literature search and expert discussion with representatives of the German Association for Palliative Medicine and the German Hospice and Palliative Care Association will inform the design of the questionnaire and DCE. The validity, comprehensibility, and feasibility of the questionnaire will be tested in a pre-test with patients and family caregivers, using the verbal probing technique [29]. To minimize the risk of response bias, the questionnaire will be formulated in a way that is generally understandable and can be completed independently by patients and relatives.

In phase 2b, semi-structured (telephone) interviews with managers from three to five selected day hospices and palliative day-care clinics in Germany will be conducted as case studies (*N* = 6–10 persons; two persons in different functions per facility). The interview guide will comprise open, stimulating questions with the aim of highlighting thematic complexes such as local networking with hospice and palliative care providers. After each interview, the guide will be reviewed and adapted. The objective of this phase will be to explore the conditions under which the respective facilities have evolved, identify the factors that promote and hinder their service provision, and elicit information on their integration into regional hospice and palliative care networks from an internal perspective. The external perspective on day hospices and palliative day-care clinics in Germany will be surveyed in phase 2c by way of focus groups [30, 31] with local actors involved in hospice work and palliative care (e.g. actors affiliated with ambulatory palliative home care, specialized palliative home care, in-patient hospices, palliative care wards, long-term care support centers, and municipalities). The results of phase 2b will be used to identify potential participants.

Using a participatory action research methodology [32], in phase 3a, 2-hour focus groups with representatives from relevant areas (e.g. the German Association for Palliative Medicine, the German Hospice and Palliative Care Association, the German College of General Practitioners and Family Physicians, the German Home Care and Nursing Society, the German Association of Towns and Municipalities, the National Association of Statutory Health Insurance Physicians, regional Associations of Statutory Health Insurance Physicians, the German Medical Association, the Association of German Cities, the National Association of Statutory Health Insurance Funds, the Association of Private Health Insurance Companies, and social and health policy) will be conducted to develop recommendations for the needs-oriented establishment and development of day hospices and palliative day-care clinics in Germany.

Finally, in phase 3b, the empirically derived recommendations from phase 3a will be agreed upon via a Delphi survey [33] with experts, including participants from phase 3a.

### Study population and data collection

Following a descriptive analysis of the initial search data from phase 1a, a heterogeneous group of palliative day-care clinic and day hospice managers (approx. *n* = 6) (from facilities of varying sizes, years of operation, urban vs. rural areas) will be recruited to participate in a qualitative telephone interview. The exact number of interviews will be determined during the course of study, on the basis of the minimum number needed to represent relevant institution-related criteria. Similarly, the final number of palliative day-care clinics and day hospices to be surveyed will be determined during this study phase. In phase 1b, the standardized written survey will be distributed to all 11 local statutory health insurance providers (AOK).

Patients (*N* = 300) and their family caregivers (*N* = 300) will be recruited in phase 2a, via six partnering internal medicine wards, acute care clinics, and rehabilitation hospitals in Lower Saxony. These participants will complete a DCE in which they will choose between hypothetical palliative care situations described by a set of attributes and corresponding levels. The underlying assumption of a DCE is that any intervention or service can be described using characteristics (attributes), and that participants will rate these differently according to the levels of each attribute provided by the relevant intervention or service (e.g. hours of care per week, activities offered). The DCE used in the present study will include eight sets, each with two alternatives. The sample size of 300 patients and 300 family caregivers is reasonable for this number of DCE choice sets, attributes, and levels. However, according to Johnson and Orme’s conservative calculation method [27, 28], only a small sample size may be required to generate significant findings. Therefore, once 20 % of the originally calculated minimum sample (*n* = 60 patients, *n* = 60 family caregivers) complete the DCE, the sample calculation will be reviewed. Notwithstanding the results of this calculation, the aim will still be to include 600 participants, in order to increase statistical power, especially for the subgroup analyses. Study eligibility will be reviewed and approved by two project study nurses, in consultation with the recruitment partners. Potential participants (patients and their family caregivers) who meet the inclusion criteria (see section “Inclusion, exclusion, and termination criteria”) will be approached by one of the project study nurses, who will personally invite them to participate, inform them of the study objectives and the relevance of the topic, and—if desired—be present when the participants complete the questionnaire, in order to answer any questions that may arise. The researchers will consult with the study nurses regularly about the recruitment process, in order to promptly identify and manage any challenges, as appropriate (i.e. by modifying the recruitment process). Six institutional partners have already agreed to assist in the recruitment process. If necessary, further recruitment partners will be integrated during the research.

In phase 2b, managers of palliative day-care clinics and day hospices (*N* = 6–10; two managers with differing responsibilities per facility) will be recruited and administered semi-structured interviews. If possible, members of the institutional founding team will be included, to enable the facility’s history to be traced. All managers should be experienced with and knowledgeable of hospice and/or palliative care structures. For this phase, the recruitment technique of snowball sampling will be utilized, as good networking between management staff is expected.

In phase 2c, local actors involved in hospice work and palliative care (*n* = 5–8 participants per group) will be questioned in focus groups. Participants will be selected according to the principle of diversity, in order to adequately represent the views of inpatient, ambulatory, general, and specialist service providers. A drop-out rate of 10 % due to illness, other commitments, and personal reasons is expected. To reach the minimum number of five participants per focus group, a maximum of eight persons will be assigned to each group.

A heterogeneous sample of key actors (*N* = 40) with sufficient influence to nationally promote the project results and recommendations will be recruited in phases 3a and b. These actors will either hold responsibility for the development of end of life care or they will work in areas that intersect with hospice or palliative care. In phase 3a, participants will be divided into a maximum of eight homogeneous focus groups of up to five persons each, in order to discuss specific topics that correspond with their areas of expertise (e.g. representatives from health insurance providers may discuss recommendations for financing). As in phase 2c, a 10 % drop-out rate is expected. In phase 3b, Delphi survey data will be collected with the aid of an online tool. Participants will be asked to indicate their level of agreement with each individual recommendation on a 4-point Likert scale, with regard to criteria such as relevance, clarity, and feasibility. Free text fields will be included for participants to provide additional comments on the recommendations.

An overlap of participants in phases 1, 2b, 2c, 3a, and 3b will promote participants’ long-term commitment to and identification with the project. The project team will enforce this through regular communication of the project status and preliminary results, as well as via individual and personal contact.

### Inclusion, exclusion, and termination criteria

Phase 2a will include patients (aged 18 years or older) who are currently hospitalized with an illness that may become palliative, in addition to their family caregivers. Patients with both oncological and non-oncological diseases (e.g. organ dysfunction or a degenerative neurological disease) will be included, provided they have sufficient physical and mental capacity to participate. Patients should not have begun palliative or hospice care, so they are able to prospectively evaluate different scenarios regarding such care. After receiving detailed information about the type, content, and purpose of the study and their participation, each patient and family caregiver will provide written informed consent to participate. Patients and family caregivers of all genders (f/m/d) and ethnic backgrounds will be invited to participate in the study.

Patients and family caregivers will be excluded from the study according to the following criteria: (1) patients are currently in a palliative or hospice care situation, (2) patients/family caregivers are not sufficiently proficient in the German language to complete the questionnaire (DCE), and/or (3) patients/family caregivers do no consent to participate in the study. Termination criteria will include: (1) significant emotional distress during participation in the study, (2) insufficient cognitive ability to complete the questionnaire (DCE), and/or (3) withdrawal of consent to participate in the study.

### Data analysis

The data analysis will aim at describing the current situation and demand for palliative day-care clinics and day hospices in Germany, and determining agreed recommendations for the establishment and development of these services.

In phase 1a, quantitative data from the online searches (i.e. a national database and general Internet search) and the palliative day-care clinic and day hospice survey will be transferred to an electronic database using IBM SPSS Statistics 26 (SPSS Inc., Chicago, IL, USA) for Windows. The data will then be analyzed using descriptive and frequency statistics. Interviews will be recorded on tape, transcribed verbatim by an external party, and analyzed qualitatively in MaxQDA (VERBI Software Consult Sozialforschung GmbH, 1989–2020), using the methodological principles of qualitative content analysis [34, 35]. Characteristics that have already been determined for the institutions will comprise an a priori category system, which will be successively expanded with the interview data. The results will provide an extended overview of the defining characteristics of these institutions, based on primary information from facility managers.

In phase 1b, the collected data on insurance providers and their contractual partners will be integrated to produce an overview of day hospices and palliative day-care clinics. Quantitative data will be evaluated using descriptive and frequency statistics.

In phase 2a, participants’ DCE data will be transferred into an electronic database. In the first step, the data will be effect coded, as follows: the binary coded care decision will represent the dependent variable; and DCE characteristics, socio-demographic data, and other personal data will be the independent variables. The data structures will be examined using descriptive analyses, and missing values will be identified. The statistical analysis will draw on random utility theory, assuming that participants will have selected the scenario (care situation) with the highest utility. Econometric methods (regression models) will be used to evaluate the data (e.g. mixed-effects models) [36]. Identification of the optimal model will be based on goodness of fit. Socio-demographic differences will be identified, as well as any relationships between preferences and illnesses (latent-class models), in order to identify potential sample subgroups with different preference weights. The results of the patient and family caregiver surveys will be compared. Statistical analyses will be carried out using R.

In phase 2b, the collected case study data will be evaluated using qualitative content analysis, according to Mayring [34, 35], as in phase 1a. The a priori topics, as defined for the interview guide, will be adopted as a category system and expanded and/or merged throughout the analysis.

In phase 2c, focus group data will be analyzed using qualitative content analysis, as in phase 2b. Coding of the collected material will be merged with the coding and a priori categories from phase 2b, to enable a systematic comparison of internal and external perspectives. The results will provide an overview of the health care services and limitations of palliative day-care clinics and day hospices. Moreover, the findings will enable an assessment of the extent to which such facilities are integrated into the local health care landscape, the challenges these facilities face, and the needs that these facilities can(not) meet.

In phase 3a, transcripts of the focus groups and notes from the feedback cards (from pin boards) will be subjected to merged qualitative content analysis, according to Mayring [37]. In advance, a priori categories will be developed on the basis of the topics addressed in the focus groups. Feedback card content will be assigned to the corresponding categories. Prior to analyzing the transcripts, relevant units of meaning and analysis will be determined. Irrelevant passages (e.g. questions and comments from the moderators and problem descriptions that do not contribute a recommendation) will not be coded. Recommendations will be paraphrased in the evaluation and summarized in the content analysis. Paraphrases with the same meaning will not be included in the category system and similar statements will be combined, in order to bundle ideas. Throughout the analysis—and on the basis of the data—the category system will be reviewed, revised, and ultimately translated into a system of higher-level categories. The final category system will be agreed upon by two scientists.

In phase 3b, recommendations from the first Delphi round that receive at least 80 % agreement from all respondents on the scale points “I rather agree” and “I fully agree” will be considered agreed. These results will be calculated by means of frequency analysis, using IBM SPSS Statistics 26 (SPSS Inc., Chicago, IL, USA). Recommendations that are lacking agreement will be revised on the basis of the free text comments from Delphi panelists and prepared for a second Delphi round. This interim analysis and adaptation of the recommendations will require approximately 2 to 3 weeks to complete. At this point, the modified recommendations will be sent to all participants who completed the first Delphi round. Again, an 80 % participation rate is expected. If necessary, depending on the data collection and analysis, a third Delphi round will be conducted. All agreed recommendations will be printed in a booklet.

**Fig. 1:**
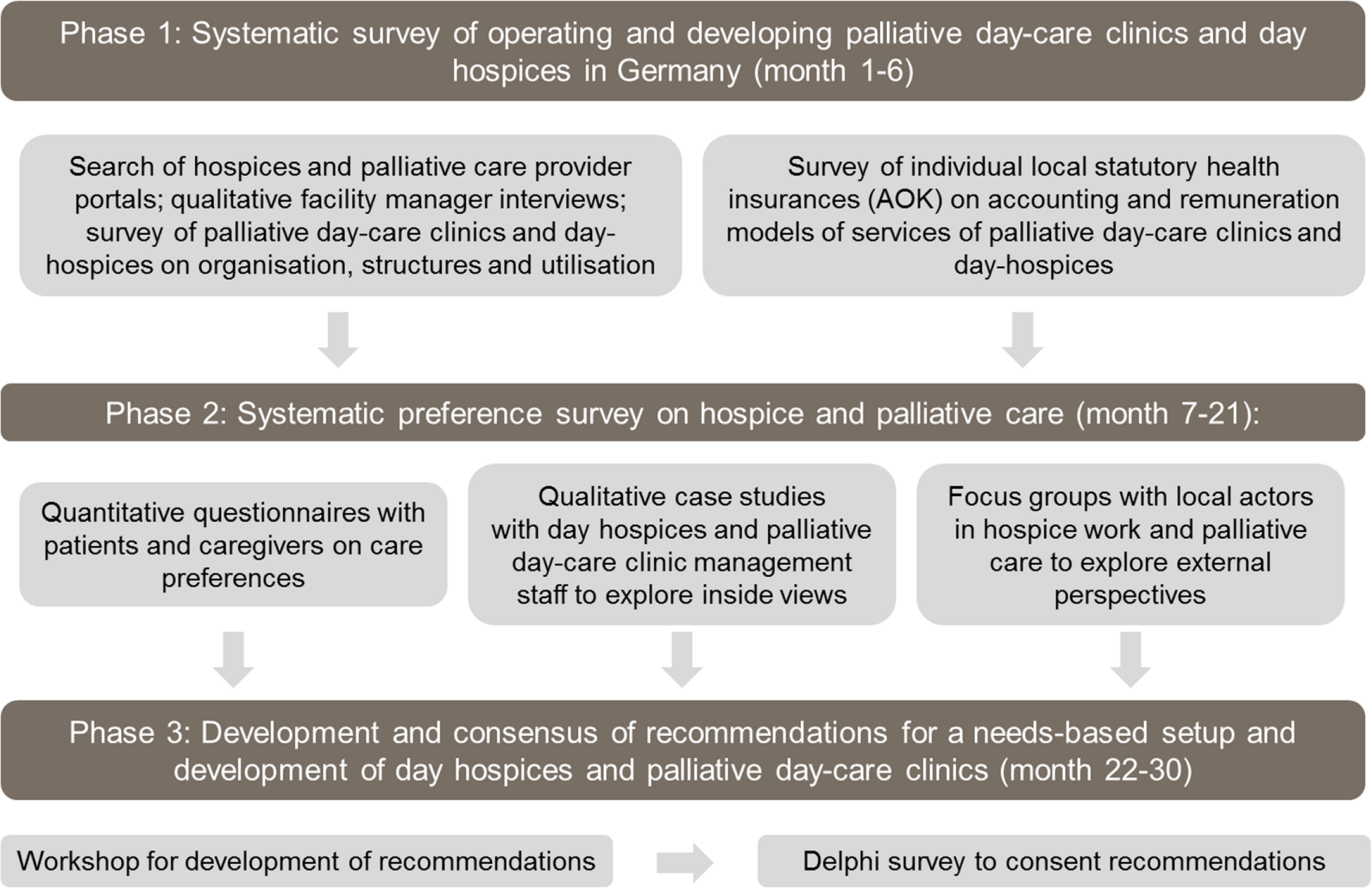
Mixed-methods study design

Figure 1 provides an overview of the mixed-methods study design across the three phases. The figure illustrates the methods used to develop recommendations for the needs-based establishment and development of day hospices and palliative day-care clinics in Germany.

### Expected results

The main expected results are: (1) an overview of the defining characteristics of day hospices and palliative day-care clinics; (2) an understanding of patients’ and family caregivers’ preferences, with regards to health care services in palliative care; (3) insight into the establishment of new facilities, factors that promote and hinder their work, and integration into regional hospice and palliative care networks, from both internal and external perspectives; and (4) a group consensus on recommendations for the needs-based establishment and development of day hospices and palliative day-care clinics. These findings will contribute to the further development of end of life care services in Germany. Moreover, the results will facilitate the effective integration of day hospices and palliative day-care clinics into wider health care structures in Germany.

## Discussion

Regarding the status of the current analysis in phase 1a, the research group is presently analyzing the search data on day hospices and palliative day-care clinics and integrating these data with feedback received from calls for feedback placed in the newsletters of the German Association for Palliative Medicine and the German Hospice and Palliative Care Association. Moreover, participant sampling criteria for the semi-structured interviews are being defined and the interview guide is being developed. At the same time, the standardized written survey for local statutory health insurance providers is being prepared.

### Study risks

Access to patients, caregivers, professionals, and health care actors can be difficult, depending on their general attitudes towards and experiences with scientific research. Hence, it may be methodologically difficult to achieve the targeted number of participants. However, the proposed case numbers should be feasible, as the study is not seeking to recruit difficult-to-reach populations and the sample sizes have been calculated in accordance with the research methods applied in the respective study phases. To assist in fulfilling the target case number for the quantitative phase 2a (with patients and caregivers), partnerships with key facilities have already been initiated (prior to the start of the study). In the event of slow recruitment, further internal medicine wards, acute care clinics, and/or rehabilitation hospitals will be approached to assist in this process. With respect to the survey in phase 2a, selection effects may arise as a result of the choice of participating hospitals and departments.

### Ethical considerations

All participating patients, caregivers, professionals, and health care actors will be informed orally and in writing about the purpose of the study, prior to their participation.

Regarding the participation of patients and family caregivers in phase 2a, no adverse events are expected, since the survey will not involve any changes in health care or interventions that may cause side effects. No further measurements and observations will be made, beyond this survey. The study nurse will personally explain the nature and purpose of the study and ask patients and family caregivers to participate. Prior to participation, the study nurse will address the methods by which confidentiality will be maintained and present the informed consent form. Participants will be asked if they understand the procedures to their full satisfaction, and they will be encouraged to ask any questions they may have. All questions will be answered by the study nurse. The survey will be administered only after the participant has signed the consent form. Each participant will receive a small token of appreciation, to a maximum value of 5.00€, in order to increase their motivation to participate. There will be no coercion, under any circumstance. Participants may experience discomfort when completing the survey, as it will ask them to reflect on hypothetical future health care options. In order to counteract this burden, the study nurses will be trained to recognize signs of distress and to provide crisis intervention, as needed.

### Data security

All personal data will be treated in accordance with the German General Data Protection Regulation. Confidentiality will be maintained by assigning an identification number to all audio recordings and questionnaires. Identification numbers and their respective participant names will be combined into a list. This list will be kept in a locked filing cabinet, separate from the interview and questionnaire data, to ensure that no link is revealed between participants’ personal data and their identification number. Consent forms will be stored separately from the interview and questionnaire data, in a locked cabinet.

For the purposes of data protection, a digital subfolder containing personal data will be assigned limited access rights. Files not kept in this folder but containing personal data will be stored in the project folder and provided with password protection. Access to the digital folders will be restricted to the researchers involved in the study. In order for the three project partners to jointly evaluate the results, the collected data will be anonymized.

The data will be exclusively analyzed with regard to the objectives stated in the project proposal. The same will hold true for any supplementary data analyses for project-related qualification. The quantitative survey data will be anonymized for the statistical analysis. The analysis of all qualitative data will be carried out in pseudonymized form (i.e. without the names of persons, institutions, or locations).

### Dissemination and implementation

To promote the accessibility and longevity of the research data and results, the research team will report the project findings in a comprehensive and transparent manner. Regardless of the findings, national and international congress presentations and peer-reviewed publications will be produced, with open access, where possible. Data files with no personal identifying information will be kept after the study completion. In accordance with the American Psychological Association Code of Ethics, Sec. 8.14, “Sharing Research Data for Verification” [38], the project leader will not withhold any unidentifiable data from other researchers who wish to verify the conclusions of the author(s). Researchers who wish to use the project data to answer new research questions must obtain prior permission from the research group and author(s).

## Conclusions

The present study protocol explains the purpose, significance, and scope of the mixed-methods ABPATITE study, as well as the study design. The empirically developed recommendations generated by this study are expected to optimize the establishment and development of day hospices and palliative day-care clinics in Germany, by ensuring they are better managed, more oriented to actual demand, and more effectively integrated into wider health care services. The results may also inform structural changes to the legal framework (e.g. to promote a framework agreement between the statutory health insurance umbrella association and health service providers). Finally, the empirically developed recommendations may be recast as a practice guide for the establishment and development of palliative day-care clinics and day hospices in Germany.

The authors’ goal of publishing the present study protocol is to promote transparency by facilitating open access to comprehensive study details that extend beyond the summary publicized in the German Clinical Trials Register. Moreover, the study protocol may act as a point of reference for the scientific community and other parties interested in the scientific and ethical aspects of the study, and prevent unnecessary duplication.

## Data Availability

Data sharing is not applicable to this article as no datasets have yet been generated or analyzed during the current study.

## Abbreviations

DCE: Discrete choice experiment.
